# A qualitative characterization of meso-activity factors to estimate soil exposure for agricultural workers

**DOI:** 10.1038/s41370-022-00484-z

**Published:** 2022-10-17

**Authors:** Sara N. Lupolt, Jacqueline Agnew, Gurumurthy Ramachandran, Thomas A. Burke, Ryan David Kennedy, Keeve E. Nachman

**Affiliations:** 1 Department of Environmental Health and Engineering, Johns Hopkins Bloomberg School of Public Health, Baltimore, MD USA; 2 Johns Hopkins Center for a Livable Future, Johns Hopkins Bloomberg School of Public Health, Baltimore, MD USA; 3 Risk Sciences and Public Policy Institute, Johns Hopkins Bloomberg School of Public Health, Baltimore, MD USA; 4 Department of Health Policy and Management, Johns Hopkins Bloomberg School of Public Health, Baltimore, MD USA; 5 Department of Health Behavior and Society, Johns Hopkins Bloomberg School of Public Health, Baltimore, MD USA

**Keywords:** Time-activity patterns, Soil ingestion, Agriculture, Macro-activity, Behavior, Meso-activity

## Abstract

**Background:**

Agricultural workers’ exposure to soil contaminants is not well characterized. Activity pattern data are a useful exposure assessment tool to estimate extent of soil contact, though existing data do not sufficiently capture the range and magnitude of soil contact in the agricultural context.

**Objective:**

We introduce meso-activity, or specific tasks, to improve traditional activity pattern methodology. We propose a conceptual framework to organize the factors that may modify soil exposure and impact soil contact estimates within each meso-activity in agriculture. We build upon models from the US EPA to demonstrate an application of this framework to dose estimation.

**Methods:**

We conducted in-depth interviews with sixteen fruit and vegetable growers in Maryland to characterize factors that influence soil exposure in agriculture. For illustrative purposes, we demonstrate the application of the framework to translate our qualitative data into quantitative estimates of soil contact using US EPA models for ingestion and dermal exposure.

**Results:**

Growers discussed six tasks, or meso-activities, involving interaction with soil and described ten factors that may impact the frequency, duration and intensity of soil contact. We organized these factors into four categories (i.e., Environmental, Activity, Timing and Receptor; EAT-R) and developed a framework to improve agricultural exposure estimation and guide future research. Using information from the interviews, we estimated average daily doses for several agricultural exposure scenarios. We demonstrated how the integration of EAT-R qualitative factors into quantitative tools for exposure assessment produce more rigorous estimates of exposure that better capture the true variability in agricultural work.

**Significance:**

Our study demonstrates how a meso-activity-centered framework can be used to refine estimates of exposure for agricultural workers. This framework will support the improvement of indirect exposure assessment tools (e.g., surveys and questionnaires) and inform more comprehensive and appropriate direct observation approaches to derive quantitative estimations of soil exposure.

**Impact statement:**

We propose a novel classification of activity pattern data that links macro and micro-activities through the quantification and characterization of meso-activities and demonstrate how the application of our qualitative framework improves soil exposure estimation for agricultural workers. These methodological advances may inform a more rigorous approach to the evaluation of pesticide and other chemical and biological exposures incurred by persons engaged in the cultivation of agricultural commodities in soil.

## Introduction

Soil can be a reservoir for pesticides, metals, per-and polyfluoroalkyl substances and other chemicals that pose risks to human health [1–4]. Exposure to soil contaminants among agricultural workers who engage in frequent, direct contact with soil is not well characterized, in part because we lack the methodology to do so. A 2022 study modeling soil and ingestion rates for the general adult population and highly soil exposure groups (e.g., construction workers and farmers/agricultural workers) predicted higher soil ingestion rates than previously published estimates derived from tracer methodology studies [5]. Incidental soil ingestion is often assumed to be the pathway that contributes the most to exposure, with the rates of ingestion assumed to vary by life stage and activity though there is little empirical evidence to support this [6].

Exposure factors are used to quantify the human behaviors and characteristics that influence exposure to an agent [7]. Those relevant in the agricultural context include the soil ingestion rate and the frequency and duration of time in contact with soil. The selection of single values or simple distributions to represent each of these factors without considering the true variation in the tasks that comprise agricultural work may mischaracterize exposure [8]. Given the intentionality and ubiquity of soil contact among agricultural workers, empirical information about the ingestion rates and frequencies and durations associated with agricultural tasks are critical to improving the rigor of measuring these exposure factors. In addition to these traditional exposure considerations, little effort has been made to include the full range of behavioral factors (e.g., use of personal protective equipment (PPE), tool use) that may influence intake or time in contact with soil. These gaps in current methods for agricultural soil exposure assessment present an opportunity to incorporate a more complete set of exposure factors to improve dose estimation for soil contaminants and better model the variability in exposure.

Activity pattern data describe the activity, frequency of activity, duration spent performing the activity, and the micro-environment in which the activity occurs [7]. They have been used in estimating individual and population exposures to air contaminants [9–11], but less so for soil contaminants [12]. These data can be used to quantify the frequency and duration of macro-activities, or broad categories of activity and the major locations in which they occur [13]. They do not, however, consider contextual variations in behaviors that may impact exposure. To date, collection of activity pattern data for soil contact has been rare and limited to the general population [7]. Consequently, these data are not informative for occupational populations like agricultural workers who, due to the nature of their work, may have higher rates of soil contact. Even among the general population, only estimates of time spent “working with soil in a garden” exist [14, 15].

In contrast to macro-activity, estimation of micro-activities involves characterizing what body part is touching a given surface for how long (i.e., duration) and how often (i.e., frequency) [13]. Micro-activity includes small-scale actions such as hand-to-mouth or object-to-mouth behaviors and has been used to inform soil ingestion rates, particularly for children [12, 13, 16]. Such data, describing rates of contact between hands, objects and the mouth, are critical for estimating both dermal and ingestion exposures [17]. Children have more frequent mouthing behaviors necessary for exploration and achievement of developmental milestones [18], and therefore, a more robust body of evidence exists for children under six years [12, 19–21]. Few micro-activity data exist for adults or for highly exposed occupational populations like agricultural workers [22].

**Fig. 1:**
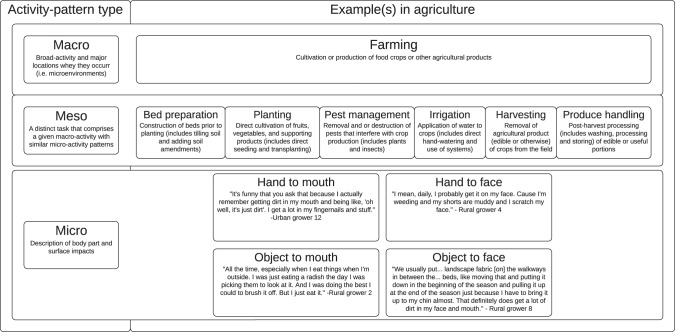
Summary of activity data types (macro- meso- and micro) and examples related to soil exposure estimation for agricultural context. This figure summarizes the types of activity-pattern data within the agricultural context. The top row defines farming as a macro-activity. The middle row defines examples of six tasks or meso-activities that emerged from in-depth interviews (IDIs). The bottom row contains examples of micro-activities described directly by growers in IDIs.

In the agricultural context where soil contact occurs via inhalation, incidental ingestion, and dermal pathways, an intermediate level of activity is needed to improve estimates of soil exposure (Fig. 1). Emphasis on macro-activity alone (i.e., farming) will not provide the nuance needed to capture the variability in intensity of soil contact across multiple routes of exposure and over time. Furthermore, assuming intake rates, or rates of micro-activity are constant across macro-activities fails to incorporate the complexity of both human and environmental factors that may impact exposure to soil.

We propose an innovative approach for improving soil exposure estimates by creating an empirical link between macro and micro-activity via a novel form of activity pattern called “meso-activity” that quantifies and describes specific tasks that serve as important predictors of soil exposure rates. Meso-activities account for task-specific nuances and provide greater resolution for how time is spent within a given macro-activity. They afford greater sensitivity to potential differences in intensity of exposure than can be achieved solely through using macro-activities. For example, farming involves a variety of distinct tasks (e.g., planting, irrigation, and harvesting) each with their own soil exposure considerations, including different patterns of micro-activity. Using crude macro-activities to estimate exposures likely results in misclassification of exposure, whereas quantification and better characterization of the factors that impact how meso-activities are conducted will account for varying intensities of exposure and thus will improve soil exposure estimation.

Qualitative research is an underutilized method in exposure science and environmental epidemiology that can aid in understanding the context (how) and motivation (why) for human behaviors that may impact exposure to environmental media. Given the formative nature of this research and the emphasis on human activities and behavior, qualitative methods are currently the most appropriate method for characterizing agricultural workers’ soil exposure. We aim to 1) identify key meso-activities routinely conducted by farmers and the factors that may impact soil exposure across and within tasks and 2) propose and demonstrate the use of a conceptual framework for integrating the environmental, activity, timing, and receptor (EAT-R) factors to guide future studies of soil exposure in the agricultural context and beyond. With a better understanding of the context in which soil exposure occurs and the factors that influence it, we can improve the design of exposure estimation tools.

## Methods

### Meso-activity framework

Our approach builds on an occupational hygiene methodology, whereby specific job titles are used as surrogates to estimate exposures among workers [23]. Epidemiologists have also employed job titles as a surrogate exposure approach in retrospective studies of occupational hazards (e.g., asbestos) [24]. Exposure assignment based on job titles, however, may be imprecise when workers with the same job title complete a variety of tasks within a single occupational setting [25]. This approach has previously been used in the agricultural context to assess pesticide exposures [26] via dermal contact and inhalation [27], but not soil exposures. In pesticide risk assessment at the US EPA, the Pesticide Handler Exposure Database (PHED) provides estimates of exposures for specific scenarios, defined by a combination of job function, chemical formulation, and level of personal protective equipment [28]. The PHED is a valuable tool which incorporates task as a key variable, while also integrating both quantitative and qualitative behavioral considerations on exposure. Agricultural work is often not limited to pesticide application, however, and given the diversity of tasks farm workers may engage in [29], we propose that a meso-activity-centered framework that focuses on better characterization of each task and pathway is a more appropriate tool for soil exposure estimation for this population.

### Participant recruitment and in-depth interviews

We used purposive sampling to identify and recruit growers via email and direct networking. Growers were eligible if they were currently a farm owner/manager, farm employee, or community gardener in Maryland, ≥ 18 years of age and had completed farm activities directly related to food production (e.g., planting, harvesting, weeding, mulching) within the past 12 months, and expected to be engaged in some agricultural tasks in the upcoming 12 months. We use the term “grower” intentionally to maintain the focus on the actions conducted by agricultural workers in this study; Using the terms “farmers” or “gardeners” evoke connections to the place (e.g., farm or garden) where the person works and may evoke misperceptions about the work environment or activities. In-depth interviews (IDI) were conducted by SL with 16 fruit and/or vegetable growers in Maryland at their farms between January and February 2020 using methods described previously [8]. All participants provided informed consent prior to the interview. All study tools and protocols were reviewed and approved by the Johns Hopkins Institutional Review Board (IRB00009866).

We used a semi-structured guide designed to gather information about farm tasks and soil contact. It began with questions asking growers to describe a typical workday and the farm operation (i.e., the distribution of the labor onsite), and included questions that asked growers to describe in greater detail the conduct of specific tasks (e.g., planting, irrigation, weeding, harvesting) mentioned during the interviews. It also included questions about soil contact (including incidental ingestion) and potential methods of increasing or decreasing soil contact (e.g., wearing personal protective equipment, typical work attire, and hand hygiene facilities onsite). Finally, the guide contained questions to solicit information about health and safety concerns experienced by growers while working onsite.

All interviews were audio-recorded and ranged from 21 to 92 min (median = 55 min; mean = 50 min). Recordings were transcribed verbatim using the NVivo transcription service and verified by one author who listened to the recordings while reading the automatically generated transcripts to verify transcription accuracy and correct typos as needed. We used the NVivo software program to facilitate the organize, coding and analysis of the qualitative data.

### Qualitative data analysis

We used a framework approach for analysis comprised of the following steps: 1) transcription; 2) familiarization with the data; 3) coding; 4) developing and working with an analytical framework; 5) applying the analytical framework [30]. We coded each transcript using a combination of inductive and deductive coding methods [31]. In the first round of coding, we developed a set of deductive codes designed to capture key concepts targeted directly in the IDI guide (e.g., farm description, farmer background, task). After coding each transcript with the set of deductive codes, we re-read all transcripts for emergent themes related to specific farming tasks mentioned. For example, we asked growers to describe a typical workday at their farm/garden. While the overall sentiment was that no day is “typical” and every day is different, every grower mentioned at least two different work activities or tasks conducted on a typical day, as well as a host of other factors that may impact which tasks were done.

We collected descriptions of what growers do at each level of activity (macro, meso and micro) while engaging in farming (i.e., the given macro-activity) and characterized the human and environmental factors that influence the task and corresponding extent of soil exposure. Figure 1 defines each of these levels and provides examples. For example, a grower may choose to wear long pants or gloves to weed in the winter when it is cold outdoors, but the same grower may choose to wear shorts and no gloves in the summer when it is warm outdoors, resulting in greater direct soil contact in the summer. Furthermore, this pattern in attire may not be consistent across all tasks (i.e., growers may wear long pants for bed preparation tasks in the summer, even when it is warm). For each of the six emergent themes (i.e., meso-activities, or tasks) (Fig. 1), we aggregated all data related to a single task (e.g., planting), and then inductively coded for factors that further describe the nature of the task (e.g., crop, technique, tool use), and the nature of soil interaction (e.g., time of day, attire worn, handwashing practices). Growers also described a variety of micro-activities (i.e., hand or object to face or mouth behaviors) that may occur within meso-activities (Fig. 1).

**Fig. 2:**
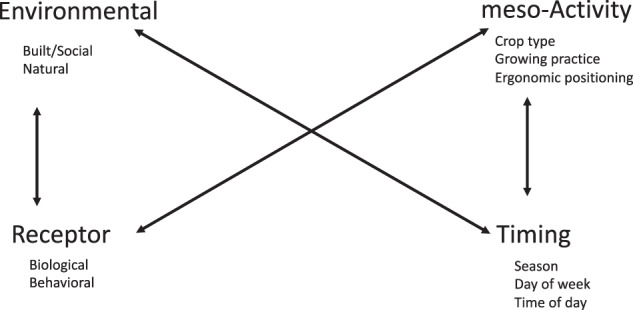
Environmental, Activity, Timing – Receptor (EAT-R) Framework describing factors impacting soil exposure in the agricultural context. This figure summarizes the environmental, activity, timing and receptor factors that may impact soil exposure in the agricultural context. The bold text indicates the four broad categories of factors identified via interviews with growers. The plain text identifies the ten sub-factors that impact soil exposures. The arrows indicate the direction of potential influences of factors on each other.

We mapped the identified factors to existing exposure science concepts (e.g., soil ingestion, time in contact with soil) [32]. For example, factors such as age, sex and attire, specific to each grower were mapped to the term “receptor.” We collapsed these into broad categories of influence (Fig. 2); for example, age and sex were grouped into “biological” factors pertaining to the receptor. Factors specific to the farm such as its location or size were mapped to the term “environment.” Some factors were collapsed into larger categories to inclusively represent the phenomena described by growers. We organized each of these ten factors into a framework comprised of four classes of factors that characterize the extent of soil exposure in the agricultural context – environmental, activity, timing, and receptor (EAT-R) factors for each task growers may engage in (Fig. 2).

### Demonstration of framework

We define each of the qualitative factors from the EAT-R framework and identify the quantitative inputs pertaining to each (Table 1). We also provide a hypothetical example of how the qualitative descriptions (informed by our IDIs) could be translated and incorporated quantitatively into traditional ingestion and dermal exposure models [33]. We also describe potential interactions between each of the factors.

**Table 1:** Description of qualitative factors in Environment, (meso-)Activity, Timing and Receptor (EAT-R) framework and summary of hypothetical quantitative impacts and interactions.

Categories in EAT-R Framework	Qualitative factors from EAT-R framework	Description of qualitative factor	Quantitative inputs impacted	Description of a hypothetical quantitative impact	Example(s) of potential interactions with other factors
**Environmental**	Natural	Climate and weather conditions may impact the micro-environment	Intake rate ^a^ Soil adherence factor ^b^	Drier environmental conditions may increase dust generation and increase dust inhalation; wetter conditions may increase adherence of soil to exposed skin	Crop type: Climate patterns impact the type of crops that are grown on a particular farm Season: Weather patterns impact the timing of particular task over a year (e.g., planting season)
	Social/Built	Farm size, characteristics and labor conditions may impact the micro-environment	Exposure factor ^a^	A farm owner/manager may set their own schedule, or the schedule/task lists of other growers	Growing practices: A farm owner/manager may establish which growing practices are used (e.g., whether/when soil amendments are applied; use of hoop houses, etc.) Crop type: a farm owner/manager decides what crops are grown, and in what quantities
**(Meso)-Activity/tasks**	Crop type	Crop type may impact the frequency, duration, or technique for a particular task	Intake rate ^a^ Exposure factor ^a^ Exposure frequency ^b^ Exposure duration ^b^	Requirements for crop cultivation may vary by crop and time of year (e.g., tomatoes are transplanted and may require trellising and pruning and are harvested in summer; leaf lettuces seeded in ground require pruning and are harvested in the spring and fall)	Season: Different crops are planted and harvested at different times of the year (e.g., tomatoes are transplanted as seedlings in spring and harvested in summer; leaf lettuces may be started in winter and summer and harvested in spring and fall)
	Growing practices	Growing practices (e.g., use of landscape fabric) or applications of soil amendments or mulches may impact the frequency, duration or techniques for a particular task; practices may vary by farm (e.g., micro-environment) as well as task or crop type	Intake rate ^a^ Exposure frequency ^b^ Exposure duration ^b^	A grower who uses mechanized equipment for tilling may disturb more soil per acre than a grower tilling by hand, or using no-till practices	Behavioral: A growing practice may be motivated by grower preference Social/Built: The use of a growing practice may be influenced by the location/characteristics of the farm (e.g., larger farms may require the use of mechanized of equipment for tilling, planting and or harvesting)
	Ergonomic positioning	Grower ergonomic position may impact proximity and intensity of contact with soil; positions may vary across and within tasks.	Intake Rate ^a^ Skin surface area ^b^	A grower who kneels or sits on the ground has more direct contact with soil than a grower who stands or bends at the waist to complete a task	Growing practices: Available equipment (e.g., mechanized equipment vs. hand tools or no tools) may influence grower ergonomic position
**Timing**	Season	Time of year (i.e., season) may impact tasks completed	Intake rate ^a^ Exposure factor ^a^ Averaging time ^b^	Different seasons may have different tasks which dominate a grower’s time	Natural: The timing of the last and first frost may impact the start and end of a “season” Social/Built: Use of season-extending equipment (e.g., hoop houses, greenhouses) may impact when particular tasks begin or end
	Day of week	Particular tasks may be conducted on particular days, and at different frequencies (e.g., daily, weekly, bimonthly)	Exposure factor ^a^ Averaging time ^b^	Different days of the week may have different work schedules or task lists; some days of the week, workers may not work on farm	Social/built: The occurrence of non-farm events (i.e., farmers markets) may impact what tasks are completed on a given day (e.g., harvesting on Friday for sales on Saturday)
	Time of day	The timing and order of tasks may differentially impact exposure	Intake rate ^a^ Soil adherence factor ^b^	A grower may irrigate soils prior to planting to reduce dust creation which may reduce inhalation rates, but may increase rates of soil adherence to skin	Natural: Weather and temperature may impact the order and timing of tasks on a given day
**Receptor**	Behavioral	Personal preferences for use of PPE, equipment, or other ways of completing a task	Intake rate ^a^ Soil adherence factor ^b^	A grower may prefer to wear gloves as personal protection, but only for particular tasks	Ergonomic positioning: A grower may prefer to squat while completing task; another may prefer to kneel Social/built: A grower’s ability to use PPE may be directly impacted by the availability of PPE and/or farm policies related to its use
	Biological	Biological characteristics of individuals may impact how they complete a task	Intake rate ^a^ Skin surface area ^b^ Bodyweight ^a,b^	A grower’s bodyweight, body composition, or strength may impact how they complete a particular task and soil intake rates for those tasks	Growing practices: A grower’s age, knowledge, or experience level may impact which growing practices they employ

^a^Input for ingestion exposure model, ^b^Input for dermal exposure model

We developed the following task-based models to demonstrate the improved precision in exposure estimation afforded by consideration of these factors in our framework (Tables S1–10). We then compare soil exposure estimates for incidental ingestion (Table 2) and dermal exposure (Table 3) pathways derived using the models informed by our framework to those generated using the traditional models for dose estimation.

**Table 2:** Summary of sensitivity analyses of ingestion exposure incorporating Environment, (meso-Activity) and Timing factors.

Description of sensitivity analysis	Summary of model parameters modified for sensitivity analysis	Description of outputs	Range of average daily doses (mg/kgBW/day)	Total average daily dose (mg/kgBW/day)
Traditional Ingestion Model	We used all defaults provided in RAGs Guidance.	Single ADD	-	4.38 × 10^−5^
	We modified the **exposure factor (EF)** to illustrate work schedules described by growers in IDIs.	Single ADD	-	9.13 × 10^−5^
Ingestion Model + meso-Activity Factors	We modified the **exposure factor (EF)** to illustrate different frequencies and durations for each task.	Task specific ADDs for 3 tasks: transplanting, harvesting, watering.	Transplanting: 2.92 × 10^−5^ Harvesting: 1.83 × 10^−5^ Watering: 3.29 × 10^−6^	5.08 × 10^−5^
Ingestion Model + meso-Activity + Timing Factors	We modified the **exposure factor (EF)** to illustrate a work schedule with different tasks over a year.	Task-season specific ADDs for 3 tasks across 4 seasons	**Spring: 3.40** × **10**^**−5**^ Transplanting: 1.46 × 10^−5^ Harvesting: 1.83 × 10^−5^ Watering: 1.10 × 10^−6^ **Summer: 3.40** × **10**^**−5**^ Transplanting: 1.46 × 10^−5^ Harvesting: 1.83 × 10^−5^ Watering: 1.10 × 10^−6^ **Fall: 1.94** × **10**^**−5**^ Transplanting: 0 Harvesting: 1.83 × 10^−5^ Watering: 1.10 × 10^−6^ **Winter: 0** Transplanting: 0 Harvesting: 0 Watering: 0	8.73 × 10^−5^
Ingestion Model + meso-Activity + Timing + Environmental Factors	We modified the **intake rate (IR)** to account for different soil conditions across different seasons.	Task-season specific ADDs for 3 tasks across 4 seasons	**Spring: 4.96** × **10**^**−5**^ Transplanting: 2.19 × 10^−5^ Harvesting: 2.74 × 10^−5^ Watering: 3.29 × 10^−7^ **Summer: 6.80** × **10**^**−5**^ Transplanting: 2.92 × 10^−5^ Harvesting: 3.65 × 10^−5^ Watering: 2.91 × 10^−6^ **Fall: 1.94** × **10**^**−5**^ Transplanting: 0 Harvesting: 1.83 × 10^−5^ Watering: 1.10 × 10^−6^ **Winter: 0** Transplanting: 0 Harvesting: 0 Watering: 0	1.37 × 10^−4^

**Table 3:** Summary of sensitivity analyses of dermal contact exposure incorporating Environment, (meso-Activity) and Timing factors.

Description of sensitivity analysis	Summary of model parameters modified for sensitivity analysis	Description of outputs	Range of average daily doses (mg/kgBW/day)	Total average daily dose (mg/kgBW/day)
Traditional dermal model	We used all defaults provided in RAGs Guidance.	Single ADD	-	1.26 × 10^−7^
	We modified the **exposure frequency (EF)** to illustrate work schedules described by growers in IDIs.	Single ADD	-	1.58 × 10^−7^
Dermal Model + meso-Activity Factors	We modified the **exposure frequency** to illustrate different frequencies and durations for each task.	Task specific ADDs for 3 tasks: transplanting, harvesting, watering.	Transplanting: 6.06 × 10^−8^ Harvesting: 3.03 × 10^−8^ Watering: 5.45 10^−8^	1.45 × 10^−7^
Dermal Model + meso-Activity + Timing Factors	We modified the **exposure frequency (EF)** to illustrate a work schedule with different tasks over a year	Task-season specific ADDs for 3 tasks across 4 seasons	**Spring: 7.88** × **10**^**−8**^ Transplanting: 3.03 × 10^−8^ Harvesting: 3.03 × 10^−8^ Watering: 1.82 × 10^−8^ **Summer: 7.88** × **10**^**−8**^ Transplanting: 3.03 × 10^−8^ Harvesting: 3.03 × 10^−8^ Watering: 1.82 × 10^−8^ **Fall: 3.64** × **10**^**−8**^ Transplanting: 0 Harvesting: 1.82 × 10^−8^ Watering: 1.82 × 10^−8^ **Winter: 0** Transplanting: 0 Harvesting: 0 Watering: 0	1.94 × 10^−7^
Dermal Model + meso-Activity + Timing + Environmental Factors	We modified the **soil to skin adherence factor (SA)** to account for different soil conditions across different seasons.	Task-season specific ADDs for 3 tasks across 4 seasons	**Spring: 2.25** × **10**^**−7**^ Transplanting: 8.66 × 10^−8^ Harvesting: 8.66 × 10^−8^ Watering: 5.19 × 10^−8^ **Summer: 7.88** × **10**^**−8**^ Transplanting: 3.03 ×10^−8^ Harvesting: 3.03 × 10^−8^ Watering: 1.82 × 10^−8^ **Fall: 1.04** × **10**^**−7**^ Transplanting: 0 Harvesting: 5.19 × 10^−8^ Watering: 5.19 × 10^−8^ **Winter: 0** Transplanting: 0 Harvesting: 0 Watering: 0	4.08 × 10^−7^

We used an iterative, stepwise approach to integrate EAT-R factors into traditional inhalation and dermal exposure models [33]. All models were designed to yield average daily doses incurred over an averaging time of one year to facilitate comparison across models. Average daily dose via ingestion was calculated using the equation:

$$Average\;daily\;dose\left( {ADD} \right) = \; 	(Concentration \ast Intake\;rate \ast Exposure\;factor) \\ 	/\left( {Bodyweight} \right)$$

where the exposure factor describes the frequency and duration of exposure for the scenario of interest divided by the averaging time. In this context, exposure factors are a term greater than or equal to 0 (no time in contact with the hazard) and less than or equal to 1 (always in contact with the hazard) that describe the fraction of time a receptor is in contact with the hazard. The average daily dose via dermal contact was calculated using the equation:

$$Average\,daily\,dose_{absorbed}({ADD} ) =	 \; ( {Absorbed\,dose_{event}} \ast Surface\,area )\,\ast Exposure\,frequency \\ 	 \ast Exposure\,duration \ast Event\,Frequency \\ 	\div ( {Bodyweight \ast Averaging\,time} ),$$

Where $$\begin{array}{l}Absorbed\,dose_{event} = Concentration \ast conversion\,factor \ast Soil\,to\,skin\,adherence\,factor\\ \ast Dermal\,absorption\,fraction\end{array}$$

Models 1 and 2 (Tables 2, 3) (Tables S1, 2) demonstrate the use of default assumptions regarding soil exposure via ingestion and dermal contact recommended in the US EPA Exposure Factors Handbook [7]. The EPA Exposure Factors Handbook contains recommended soil ingestion rates, estimated time in contact with soil, bodyweights, and anthropometric data pertaining to dermal exposure from the scientific literature and national surveys. These recommendations are frequently used to inform exposure assessments for contaminated lands and derive public and occupational health guidance values for contaminants in soil. Though the Exposure Factors Handbook does not have recommendations specific to agricultural scenarios, we chose inputs (Tables S1–10) for these models to most closely align with an agricultural exposure scenario. Models 3 and 4 (Tables S3, 4) illustrate the integration of meso-activity and yield average daily dose estimates (for ingestion and dermal exposure) for each task. In models 5 and 6 (Tables S5, 6) we incorporate timing factors to demonstrate the seasonal nature of agricultural work and account for the differential conduct of specific tasks across seasons. Models 7 and 8 (Tables S7, 8) show how environmental factors (e.g., differences in soil moisture attributable to different weather conditions) may impact soil exposure. Specifically, in Model 7 we differentially vary the intake rate across seasons for the ingestion model. In Model 8 we vary the skin to soil adherence factor across seasons for the dermal model. In models 9 and 10 (Tables S9, 10) we illustrate the impact of person-specific receptor factors (e.g., sex-specific body weights and differences in seasonal preferences for attire) by modeling exposure for two hypothetical growers of different ages and sex and different behavioral preferences.

## Results

### Study population

Most growers interviewed were female (*n* = 9), working full time (≥ 35 h per week) (*n* = 10) and working in Baltimore city (*n* = 9) (i.e., hereafter described as “urban”; growers working outside of Baltimore are described as “rural”) [8]. When growers had previous experience working operations both inside (urban) and outside Baltimore city (rural), we classified them according to the location of their operation at the time of the interview.

### Meso-activities and modifying factors

Six distinct, routine meso-activities, or tasks essential to fruit and vegetable production, were identified as emergent themes: bed preparation, planting, irrigation, harvesting, pest management and produce handling. In addition to these tasks (typically completed outdoors) essential for fruit and vegetable production, several growers also described tasks conducted indoors such as office work, (e.g., ordering supplies, developing crop plans, communication with customers) and retail related tasks (e.g., transporting, delivering, and selling produce). Animal care (e.g., chickens, pigs) was an additional task mentioned by six growers, though only four operations were raising animals at the time of the interview. Because these tasks were less often discussed and were less likely to involve soil contact, the data were not sufficient to include in the conceptual framework. In addition, growers also described a range of micro-activities, or non-dietary ingestion behaviors (hand to mouth; object to face) that may occur while engaging in each of these meso-activities and may also contribute to soil exposure. Illustrative quotes describing each of these pathways are included in Fig. 1 and were also considered by the authors in identifying the factors and deriving the conceptual framework.

### EAT-R framework

Growers described 10 factors as influencing how or why they might complete a farming task, or how the extent of soil contact varies while completing the task. We were interested in factors that may impact how soil contact occurs while a given task is completed as well as upstream factors that may impact how and/or how often a task is completed in each context.

All factors were grouped into four classes: environmental, activity, timing and receptor, (EAT-R) each describing which aspect of the exposure scenario is impacted by the factors (Fig. 2). Environment refers to where the exposure occurs; activity refers to the task and how it is done; timing refers to when and how often a task is done; receptor refers to who is exposed.

## Environmental factors

Microenvironments are “surroundings that can be treated as homogeneous or well characterized in the concentrations of an agent” [32]. In our framework, environmental factors include both social/built and natural factors that may impact the conditions or attributes of the immediate surroundings of a grower while working on the farm. We identified two natural factors (climate and weather) and three social/built factors (farm size/type; technology/facilities and workforce).

### Natural factors

We observed that the geographic location of a farm has significant implications for the on-farm conditions of soil contact. For example, where a farm is located may impact broad climatic conditions that may dictate the length of the growing season and which crops can be grown. Growers described how both short-term weather – and long-term climatic patterns impact soil exposure. One grower described the impact of short-term weather conditions on soil exposure:

Urban grower 1: “I think weather has a huge impact on just how much soil contact you’re getting… But if I were to go outside right now, I could dig a hole. I could do a lot of tasks without really getting meaningfully dirty because of the environmental conditions. Now, […] if we’re going through a drought, like if it’s really dry and everything’s dusty and you can’t go outside without getting dusty and dirty and just it’s from […] being kicked up with minimal disturbance. If it’s raining, it’s kind of the opposite issue, but it’s the same thing.”

In addition to short-term weather patterns, several growers also discussed the long-term impacts of climate and how growing practices have shifted because of climate change. For example:

Urban grower 11: “We do still have to have elevated beds because of the torrential rains we get now. We didn’t used [to]. Yeah, farming’s changed a huge amount in twelve years. Huge.”

### Social/Built factors

Farm size/type may also impact soil contact while engaging in each task. We interviewed growers producing fruits and vegetables on plots in urban and rural areas of Maryland, ranging from less than 19–607,028 square meters. We observed important differences in how growers describe their farms and even quantify the size of their farms. For example, one grower used type and volume to describe the composition of crops sown as well as differentiating by whether the crop was direct seeded or transplanted.

Rural grower 14: “Well, it’s funny. If you’re saying type of crops, it’s half and half [i.e., half direct seeded; half transplanted]. OK, but if you’re saying volume of crops, it’s more transplants.”

Another farmer used dollar sales and square acreage to describe the size of their farm:

Rural grower 9: “That’s an interesting question. In terms of dollar sales or in terms of square footage?… Well, most farms talk about acreage. So we’ll talk about that.”

Related to farm-size/type, the size of the workforce and site policies and procedures for distributing labor will impact how soil contact is distributed among workers onsite. Some operations were staffed by a single farmer, while others had several employees and/or volunteers:

Rural grower 2: “Last year, I had two full time people that worked about thirty-five hours a week. And I have several volunteers and I have usually one to two part time people do anywhere from 10 to 20 hours a week.”

A grower in a leadership position, such as farm owner or manager in the farm business who is responsible for a greater diversity of tasks, may have the ability to vary, postpone or even avoid by delegation, specific tasks that may be particularly soil intensive. A grower with less autonomy may have less control and specialize in a limited number of tasks, including those that may be more soil intensive. A sole proprietor who is responsible for all tasks may have the greatest soil contact, attributable to the fact that completing tasks individually may take more time and without assistance.

At all farms, the technology and facilities available on site may have important implications for both the schedule of tasks and nature of soil contact within each task. Most obviously, use of mechanized equipment may not be possible on plots less than 500 square feet. Some growers invest in onsite technology such as high tunnels and/or greenhouses to extend the growing seasons which may introduce new tasks or extend the frequency of existing tasks beyond the natural growing season. In addition, the availability of restroom and handwashing facilities may impact how often it is feasible for growers to wash hands while working (e.g., after each task, as needed, or only at the end of the workday).

## Activity factors

While a task may be described as an action completed by a grower to advance the production of food, activity factors describe further variation in how and/or why a particular task is completed. Such considerations are important and warranted given the qualitative differences in the amount of soil growers encountered even within the same task. We identified three key factors that impact soil exposure within a given task: crop type, growing practices and ergonomic positioning.

### Crop type

Our conversations with growers revealed that soil exposures may also vary within a specific task, depending on the crop type. For example, harvesting tomatoes or other fruiting plants may require minimal soil contact, whereas harvesting sweet potatoes or other root crops which are completely immersed in soil may require more soil contact. One grower made a point to emphasize the soil contact required for harvesting sweet potatoes:

Rural grower 14: “So you’re digging in the dirt. You’re digging for gold. That is a massive, massive dirt experience.”

### Growing practices

Growers described a range of growing practices within a given task that may impact soil exposure. Variability in growing practices may be driven by crop type (e.g., some plants are direct seeded, others are transplanted as seedlings) or the tools and technology available onsite. For example, as planting was discussed, most growers made a point of differentiating between direct seeding (i.e., planting seeds) and transplant (i.e., planting seedlings) methods of planting. Of note, these variations are not necessarily mutually exclusive as some growers may opt for both methods (i.e., starting seeds in a greenhouse, then transplanting the seedlings when more mature) within the same season for the same crop.

Within a given method of planting, growers described a range of tool use that may impact soil contact. For example, one rural grower described his/her method for directly planting seeds with low mechanization (hand-tools only):

Rural grower 2: “It’s like a piece of wood, a piece of metal that goes down to a point with a handle and you make a hole in the ground. And then you go through and put the plant in. So we’re a small farm and we do a lot of things by hand, which I’ve already said. […] It’s by hand on our hands and knees, sometimes bent over. Sometimes you can straddle the row like this.”

Another rural grower described seeding using an intermediate (non-hand, non-engine based) level of mechanization:

Rural grower 7: “I just have a little push seeder. And depending on the seed size and spacing, you just change some plates around and then that’s it.”

Finally, a third rural grower described a highly mechanized process for sowing transplants:

Rural grower 9: “We use what’s called a water wheel transplanter. It’s a […] wheel assembly that is pulled behind a tractor. It has large tanks that have water in it. And it has two seats for people that are riding on the back of that and it holds trays of transplants. And that piece of equipment and the tractor straddles a bed and there’s wheels that poke holes. And they [the workers] take the transplant and they stick the transplant in the hole.”

Related to bed preparation, growers described a range of different techniques for tilling. One grower described both using less mechanized techniques for tilling (using hand tools) and more mechanized techniques (using tilthers) for the same bed preparation task:

Rural grower 2: “So the way we prep a bed is with a broad fork. So we go down. We have mostly 100-foot beds. We go down the beds with the broad fork and then we rake. If we’re putting amendments on, we put the amendments on. We rake in the amendments. And then I have a tilther […] that’s like a little mini tiller. And it just really turns to the top one inch to half inch of the soil. So it mixes in the amendments.”

Some growing practices such as planting cover crops may impact soil exposure. Ten growers discussed the cultivation of cover crops, which are often non-commodity or specialty crops included in the crop rotation to improve soil health. Most commonly, growers described planting cover crops for the fall or winter to improve soil health. A rural grower described an additional benefit of cover cropping is, “to keep the soil in this place,” as well as to prevent erosion and limit the amount of soil exposed and subsequently growers’ exposure during the non-growing season. When assessing soil exposure associated with planting and bed preparation tasks, it may be tempting to overlook the cultivation of cover crops and focus exclusively on edible or cash crops; however, growers described a variety of labor and time intensive processes for cultivating and then tilling cover crops. At one end of the spectrum growers described a highly mechanized, high tilling scenario:

Urban grower 8: “Plant[ing] a cover crop in the fall, which usually involves the disc harrow, a harrow with a disc implement with the tractor and planting cover crops, each with a tractor mounted seeder, either like grain drill or just something that flings a seed around in the air and broadcast it. In the spring, we mow the cover crop with a mower and we stay in the field, which is like a big tiller.”

A rural grower described a similar process in terms of mechanization but with an emphasis on minimizing soil disturbance through no-till techniques:

Rural grower 9: “We rent a large piece of machinery from the soil conservation district and that is how we put new seeds for cover crops in the ground. It doesn’t do what we call soil disturbance. There’s no ploughing. There’s no tilling of the soil. It’s essentially a slit in the ground and a seed is placed in there and covered up. It’s why it’s called no tillage, no till tools.”

### Ergonomic positioning

While growing practices, the type of crops and the mechanized tools available may impact the nature of soil exposure, the positioning of the farmer while engaging in the specific task may have significant implications for soil exposure. Most commonly, several growers described hand weeding (as a type of pest management) being done on hands and knees:

Rural grower 7: “Sometimes if you did a really crappy job and you need to go through and literally hand weed the whole thing, you’re on your hands and knees and you have, you know, mud in your knees you have mud on your hands and yeah, definitely.”

Another grower described how positioning impacts incidental ingestion of soil and dermal contact of the face and soil during planting:

Rural grower 8: “But during planting, it definitely can happen occasionally because you’re so close, your face is so close to the ground.”

Growers generally described minimal direct contact with soil while riding a tractor, but they described increased dust exposure due to greater disruption of soil by the mechanized equipment. While soil contact may be a factor in determining a growers’ positioning, ergonomic considerations may also be an important factor in determining how a grower positions him/herself:

Rural grower 8: “It can be a variety of ones. But my most comfortable position is bending over and like keeping my legs straight and just bending my back at practically a 90-degree angle.”

Urban grower 6: “I try to use implements as much as I can to save my back. I’m a big believer in the stirrup hoe. I like that tool a lot.”

## Timing factors

Timing factors are variables that describe when the tasks are completed and exposure events occur. The frequency (i.e., how often) and duration (i.e., how long) of each task are important considerations traditionally used to time-weight exposure or time in contact with an agent. We observed variation in both factors and have included them in our conceptual framework. Most growers reported that there is not a “typical workday” and reported a length of 1 to 17 h per day of work; the number of days worked per week ranged from 3 to 7. In addition, growers reported that season, day of week and time of day contribute additional variability and may dictate when a specific task is completed.

### Season

Growers described a “growing season” covering the months of May through September, or November, depending on the farm. The length of time growers work during the growing season was generally greater than the amount of time in the off season:

Rural grower 4: “[I] literally work from sunup to sundown. Now, I might come in and take an hour lunch or I might take a little break in the afternoon because I’m tired.”

### Day of week

Another described a typical schedule by day of the week with a rigid schedule dictated by the external obligations related more broadly to the business:

Rural grower 2: “Because … we have [Community Supported Agriculture] (CSA) deliveries on Tuesday and CSA pickup on the farm on Tuesday. So therefore, Monday has to be harvesting. When we work with the food hub, restaurant deliveries go out from the food hub on Tuesday mornings and Thursday mornings, which means Monday morning and Wednesday morning become harvest days. We have restaurant orders. Tuesdays are deliveries and farm stand. That’s when we have our farm store open for business.”

### Temporality

One urban grower mentioned timing specific tasks according to temporality, specifically, the time of day– for example, harvesting greens early in the morning, when they are still wet with dew and at their peak. In addition to these factors, several rural growers described having a crop plan that guided when tasks are conducted:

Rural grower 9: “I have a plan of the day and a plan of the week. That, again, is driven by the crop plan, but it’s also driven by observations of the field and what’s going on with the weather conditions.”

The lack of uniformity in typical workday suggests a need for more refined and systematic investigation of when specific food production-related tasks may occur.

## Receptor factors

Receptor factors are variables that describe the person(s) conducting the tasks and incurring exposure. Receptor factors include relatively static biological characteristics of the receptor (e.g., age, sex, and/or anthropometrics, etc.) as well as modifiable behavioral characteristics such as experiences and personal preferences.

### Biological factors

Outside of the information collected at the time of interview (e.g., sex, age), growers did not generally discuss their own biological factors in the context of our interviews, which may be indicative of these factors’ relatively low salience in the growers’ perceived importance in the context of soil exposures. Though growers did not extensively or routinely discuss physiological factors as impacting their soil exposure, we added these to our model given the centrality of these figures in the existing paradigm of exposure factors, especially those related to dermal contact.

### Behavioral factors, including use of PPE

Experience was identified as one receptor factor that may impact how a given task is completed. For example, one urban grower described specific tools she uses when working with less experienced volunteers when transplanting:

Urban grower 1: “If I’m working with volunteers or someone who doesn’t really know, I’ll try to have some form of device. Usually it’s just like a rod that has like spacing worked out so that they know like that’s three inches, six inches. And so that stays consistent.”

Two rural growers described requiring additional, if informal, training for workers to operate tractor equipment. Depending on the task, using a tractor may result in less soil contact. Three rural growers mentioned additional experience was needed to apply pesticides onsite – either with a formal class or via instructions from a supervisor. Thus, depending on experience, certain tasks may or may not even be assigned to a given grower.

Related to experience are specific behavioral preferences for how a task is done.

An example of a preference might be the decision to wear a particular type of clothing or form of personal protective equipment while engaging in a particular activity, or whether to use a particular tool. For example, a grower may choose to wear gloves while weeding, but not while planting; another grower may do the opposite.

We consider PPE a behavioral intervention to mitigate exposures and thus within our framework consider the use of PPE a behavioral factor. Previous studies of PPE to mitigate injuries [34] and sun exposure risks [35] in agriculture and other industries [36] are firmly rooted in a behavioral context. Building on our previous work [8], we urge the consideration of not only the use of PPE in soil exposure models for agricultural workers but also the growers’ attitudes and motivations pertaining to its use. These behavioral considerations are critical for characterizing not only the use of PPE but also its correct use its use and the duration and consistency of its use across different meso-activities and environments.

The frequency of other non-dietary ingestion behaviors, (i.e., smoking or sampling produce) may impact soil contact. Smoking onsite may be a relevant behavior for estimating exposure to soil contaminants, not directly due to smoking, but due to the act of bringing the hands (which may be covered in soil or dust) close to the face, which may increase incidental soil ingestion. Sampling, or tasting produce to assess quality in the field, was also a behavior described by several farmers:

Rural grower 2: “All the time, especially when I eat things when I’m outside. I was just eating a radish the day I was picking them to look at it. And I was doing the best I could to brush it off. But I just eat it.”

## Interactions between factors

In addition to identifying the factors that may impact soil exposure among agricultural workers, our conversations also helped us elucidate how some of the individual factors identified may interact to impact soil exposure. For example, one grower described two timing factors (season and day of week) impacting the frequency and duration of tasks done on site:

Rural grower 7: “So a typical workday really depends on the day of the week, depending on time of the year.”

Another example may be the relationship between the level of mechanization and number of workers onsite. For example, the water wheel transplanter is a highly mechanized tool that connects to a tractor, and as described by several growers, allows three workers to complete a transplanting task much more efficiently than if ten workers transplanted with no mechanized assistance. Thus, there is a possible tradeoff between the number of workers exposed and the extent of soil contact. Without additional probing, observation, or quantitative methods, we are unable to determine from this explanation alone how these factors impact soil exposure, i.e., the extent to which the factors would impact soil exposure and whether the season and day of week factors would increase or decrease soil exposure. Furthermore, we are unable to determine whether this interaction between factors is consistent across all observed tasks or only a few.

In addition, different classes of factors may interact to impact soil exposure within a particular task. For example, one grower described how a combination of activity (crops, growing practices, ergonomic positioning) and environment (farm size, technology and facilities) factors impacted how planting occurred:

Rural grower 16: “So probably the time that I was that I would have the most soil contact and get soil in my mouth and on very strange parts of my person was planting peppers, tomatoes and eggplants because generally at that scale even and even at the larger, larger rural scale, it was on my knees planting with a hand trowel, digging a hole out, planting the plant and then kind of scooting down and moving down that way. It wasn’t until we scaled up, we’re using it, transplanter it to put stuff in like that. But before that, the whole time was like, you planting tomatoes like that tall and burying it that deep. So that required getting in and digging out by hand.”

## Application of the framework

This section describes the series of exposure calculations and sensitivity analyses we conducted to demonstrate (quantitatively) the hypothetical influences of different qualitative factors described by growers on soil exposure. Our current study was not designed to derive quantitative estimates of the relative impact of each of the qualitative factors we identified and described. In some cases, there is little existing evidence to directly inform the quantitative adjustments we make. In those cases, we use our professional judgement to assign quantitative values to qualitative factors in the framework to illustrate their potential impact on exposure estimates. All assumptions and parameters are stated in the supplemental material (Tables S1–10). Summaries of these sensitivity analyses are provided in Tables 2–4.

**Table 4:** Summary of sensitivity analyses of ingestion and dermal contact exposure incorporating Environment, (meso-Activity), Timing and Receptor factors.

Description of sensitivity analysis	Summary of model parameters modified for sensitivity analysis	Description of outputs	Grower A Range of average daily doses (mg/kgBW/day)	Grower B Range of average daily doses (mg/kgBW/day)
Ingestion Model + meso-Activity + Timing + Environmental + Receptor Factors	We adjusted the **body weights (BW)** and the **intake rates (IR)** across tasks and seasons independently across Grower A and B.	Task-season specific ADDs for two growers (Grower A and Grower B)	**Spring: 5.50** × **10**^**−5**^ Transplanting: 2.43 × 10^−5^ Harvesting: 3.03 × 10^−5^ Watering: 3.64 × 10^−7^ **Summer: 9.72** × **10**^**−5**^ Transplanting: 3.24 × 10^−5^ Harvesting: 6.47 × 10^−5^ Watering: 1.21 × 10^−7^ **Fall: 2.14** × **10**^**−5**^ Transplanting: 0 Harvesting: 2.02 × 10^−5^ Watering: 1.21 × 10^−6^ **Winter: 0** Transplanting: 0 Harvesting: 0 Watering: 0	**Spring: 2.01** × **10**^**−5**^ Transplanting: 8.80 × 10^−6^ Harvesting: 1.10 × 10^−5^ Watering: 2.83 × 10^−7^ **Summer: 3.40** × **10**^**−5**^ Transplanting: 1.51 × 10^−5^ Harvesting: 1.89 × 10^−5^ Watering: 9.43 × 10^−8^ **Fall: 1.67** × **10**^**−5**^ Transplanting: 0 Harvesting: 1.57 × 10^−5^ Watering: 9.43 × 10^−7^ **Winter: 0** Transplanting: 0 Harvesting: 0 Watering: 0
			Total average daily dose: 1.74 × 10^−4^	Total average daily dose: 7.08 × 10^−5^
Dermal Model + meso-Activity + Timing + Environmental + Receptor Factors	We adjusted the **body weights (BW)** and **the skin surface area available** across tasks and seasons independently across Grower A and B.	Task-season specific ADDs for two growers (Grower A and Grower B)	**Spring: 3.97** × **10**^**−9**^ Transplanting: 1.37 × 10^−9^ Harvesting: 1.62 × 10^−9^ Watering: 9.74 10^−10^ **Summer: 3.01** × **10**^**−9**^ Transplanting: 1.16 × 10^−9^ Harvesting: 1.16 × 10^−9^ Watering: 6.94 × 10^−10^ **Fall: 1.22** × **10**^**−9**^ Transplanting: 0 Harvesting: 6.12 × 10^−10^ Watering: 6.12 × 10^−10^ **Winter: 0** Transplanting: 0 Harvesting: 0 Watering: 0	**Spring: 2.25** × **10**^**−7**^ Transplanting: 8.66 × 10^−8^ Harvesting: 8.66 × 10^−8^ Watering: 5.19 × 10^−8^ **Summer: 6.78** × **10**^**−8**^ Transplanting: 2.61 × 10^−8^ Harvesting: 2.61 × 10^−8^ Watering: 1.56 × 10^−8^ **Fall: 8.94** × **10**^**−8**^ Transplanting: 0 Harvesting: 4.47 × 10^−8^ Watering: 4.47 × 10^−8^ **Winter: 0** Transplanting: 0 Harvesting: 0 Watering: 0
			Total average daily dose: 8.02 × 10^−9^	Total average daily dose: 3.82 × 10^−7^

### Traditional occupational exposure model

In our first set of sensitivity analyses, we modified the exposure factor (for ingestion) and frequency (for dermal contact) parameters using information about typical number of hours worked (per day and per week) as provided by growers who participated in this study (Tables S1, 2). Using a traditional model for estimation of soil exposure from the US EPA Risk Assessment Guidance for Superfund with default assumptions from the Exposure Factors Handbook as our base model allows general estimates of average daily doses of contaminants but probably results in a mischaracterization of exposure. The default exposure factor used in the base occupational model assumes a worker that is exposed over 8 hours of work per 24 hour period, 5 days of work per 7-day week and 50 weeks of work per calendar year (equaling 0.16). Qualitative data from our in-depth interviews with growers provided anecdotal evidence that is common for them to work for at least 10 hours per 24 hour period and for 6 days per 7-day week. As sensitivity analysis 1 we recalculated the exposure factor with these data to obtain an exposure factor of 0.34 (Table S1). Use of the base occupational model to calculate the exposure (0.16–0.34), in this hypothetical scenario, would result in an under estimate of soil exposure by 50% (4.38 × 10^−5^ vs. 9.13 × 10^−5^ mg/kgBW/day) (Table 2).

### Exposure model incorporating meso-activity factors

In our second set of sensitivity analyses we estimated exposure for three different tasks (i.e., transplanting, harvesting, and watering) by modifying the exposure factor (for ingestion) and frequency (for dermal contact) parameters for each task-dose calculation. Estimating dose for each activity (or task) independently allows for variability in intensity of exposure across tasks. In sensitivity analysis 3 (Table 2; Table S3), the overall dose estimate for ingestion exposure was only about 16% greater than in sensitivity analysis 1, but the transplanting task contributed almost ten times more to ingestion exposures than the irrigation task. This finding highlights the benefit of this approach for public health practice in that it identifies the task(s) that may be most appropriate for intervention to reduce exposure, if necessary. In sensitivity analysis 4 (Table 3; Table S4), all task specific dose estimates were within the same order of magnitude (i.e., 3.03 × 10^−8^–6.06 × 10^−8^ mg/kgBW/day) but were an order of magnitude less than the base dermal exposure estimate (Table S4).

### Exposure model incorporating meso-activity and timing factors

In our third set of sensitivity analyses, we estimated exposure for three different tasks across four seasons (i.e., spring, summer, fall, winter) by modifying the exposure factor (for ingestion) and frequency (for dermal contact) parameters for each task-season dose calculation. Our conversations with growers and exposure modeling verified the importance of seasonality and the timing of tasks as critical sources of variability in growers’ tasks and subsequent soil exposure. Sensitivity analyses 5 and 6 (Tables S5, 6) demonstrate the variability in seasonal exposures, when considering season-specific frequencies and durations. Seasonal ingestion exposures range from 0–3.40 × 10^−5^ mg/kgBW/day, with an annual total of 8.73 × 10^−5^ mg/kgBW/day (Table 2; Table S5); seasonal dermal exposures range from 0 to 7.9 × 10^−7^ mg/kgBW/day with an annual total of 1.9 × 10^−7^ mg/kgBW/day (Table 3; Table S6). Though these sensitivity analyses do not directly account for this, additional investigation regarding how (dermal in particular) exposure varies over a 24 hour period in relation to the frequency and temporality of handwashing pertaining to specific tasks is also needed.

### Exposure model incorporating meso-activity, timing and environmental factors

In our fourth set of sensitivity analyses, we estimated exposure for three different tasks across four seasons and used our best professional judgement to vary the intake rates (for ingestion) and soil to skin adherence factors (for dermal contact) parameters across seasons. Our modifications were rooted in the assumption that changes in environmental conditions may impact exposure rates. For example, dryer conditions may produce more dust, increasing ingestion rates, or wetter conditions may result in greater adherence of soil particles to exposed skin. In sensitivity analyses 7 and 8 (Tables S7, 8) we further consider how environmental factors may influence intake related parameters (e.g., intake rates and soil adherence rates.) For example, increased precipitation in the spring and fall seasons may result in a higher moisture content in soils. Soils with greater moisture may adhere more strongly to skin, translating to a greater soil to skin adherence rate for dermal exposures. Given the lack of empirical data or recommended defaults in the Exposure Factors Handbook, we modified the soil to skin adherence factors from the default of 0.07 to values ranging from 0.07 (for winter and summer) to 0.2 (for spring and fall). These hypothetical values were derived from the authors’ best professional judgement (Tables S7, 8). Moisture in the air and soil during the shoulder seasons may reduce the presence of dust and decrease exposure via the ingestion and inhalation pathways. Local weather conditions (e.g., precipitation on a given day) may also alter whether a task is completed on a particular day or postponed until weather conditions improve.

### Exposure model incorporating meso-activity, timing, environmental and receptor factors

In our fifth set of sensitivity analyses, we estimated exposure for two hypothetical growers, who completed three different tasks across four seasons. Our modifications included selection of different body weights appropriate to growers of different ages and sex. We also modified intake rates (for ingestion exposures) and skin surface available parameters (for dermal exposure) by season and task to account for variability in tool use, personal habits, and grower attire across seasons. Sensitivity analyses 9 and 10 (Tables S9, 10) illustrate the impact of biological factors specific to the grower (e.g., body weight) and behavioral factors related to seasonal attire preferences and the unique conduct of specific tasks. In these sensitivity analyses we model exposure for two growers demonstrating the range of experiences described in the IDIs. Grower A is a female grower in her 20 s who often samples produce while harvesting, only wears gloves for transplanting in spring and sometimes works barefoot/with sandals in spring and summer. Grower B represents a male grower in his 40 s who samples produce while harvesting, uses a tractor and other mechanized equipment frequently (esp. for transplanting and harvesting tasks) and wears gloves at all times. Between these hypothetical, but exemplary growers, dermal exposures differed two orders of magnitude (Grower A = 8.2 × 10^−9^ mg/kgBW/day vs 3.82 × 10^−7^ mg/kgBW/day) and ingestion exposures differed by one order of magnitude (Grower A = 1.74 × 10^−4^ mg/kgBW/day vs. Grower B = 7.08 × 10^−5^ mg/kgBW/day) (Table 4).

We found that each iteration of our model to include more of the factors identified in the EAT-R framework demonstrated the rigor and nuance needed to support estimates of soil exposure for agricultural workers. This hypothetical example shows how our framework is compatible with existing dose estimation models, and demonstrates the key benefit of our framework, specifically, our ability to capture variability in exposure related to a range of environmental, activity, timing and receptor factors.

## Discussion

To date, attempts to characterize agricultural soil contact lack the rigor and nuance needed to inform exposure and risk estimation. Our conversations with growers identified a wide variety of factors that may impact agricultural soil exposure. We identified ten specific variables or characteristics that may impact soil exposure in the agricultural context and grouped them into four broader classes of factors. Our framework is embedded within the existing activity pattern methodology for macro-activities used to estimate time in contact with a potential medium/contaminant and provides a more targeted approach quantifying the frequency, duration, and intensity of soil exposure across different farming tasks, or meso-activities. In our framework, we identify the factors that may impact the frequency, duration and intensity of soil exposure. We demonstrate how these qualitative factors may be translated to quantitative inputs used in dose estimation equations. Compared to our more rigorous estimates using this meso-activity framework, we found that the traditional approach may significantly underestimate or overestimate (depending on the nature of the work) soil exposure. Broader investigations of agricultural workers to build a database of their meso-activity associated exposure factors are needed to advance and finetune soil exposure estimation in agriculture. Future studies are needed to empirically assign quantitative values to and mathematical relationships between each of these parameters. A key strength of our framework is its applicability to all pathways of soil exposure – (incidental) ingestion, inhalation and dermal contact. Direct dermal contact with soil and ingestion and inhalation of soil and dust is possible during all the activities described by growers.

This framework aids in estimation of soil exposure by characterizing key factors that impact soil exposure for each meso-activity. Using indirect measurement tools (i.e., questionnaires) exposure scientists could collect activity pattern data to construct mathematical models to improve soil exposure estimates. For each meso-activity, or task, growers could be queried on the frequency, duration, and timing of the task. This can be estimated easily by growers themselves, or a review of growers’ crop plans verified via direct observation. Next, exposure scientists could query growers about the nature of the task and how it is done. For example, the task of planting seeds could be characterized with answers to the following questions: “what kind of seeds were planted? how many seeds were planted? how were they planted? how was the grower positioned while planting? were any tools used?” Next, exposure scientists could query growers about their own behavioral and biological characteristics that may impact soil exposure. Finally, querying growers about the characteristics of the farm, or location where the activity occurs would yield key insights into micro-environmental conditions that may influence exposure.

Our qualitative task-centered investigation, which is more common in occupational exposure assessment [23, 37] than risk assessment, provides a more nuanced approach to soil exposure assessment for the agricultural sector than can be obtained from previous surveys which consider agricultural work as a single task [38]. Because these activities are intentional, quantification of the frequency and duration of these activities through traditional self-reported, survey methods (e.g., time-activity diaries and questionnaires) is possible. While the extent of soil contact may be modified by a variety of quantitative (e.g., frequency and duration) and qualitative (e.g., mechanization and technique) activity-specific factors, this framework provides a baseline for further investigation of the nature of soil exposure in agriculture.

Though we interviewed only fruit and vegetable growers in Maryland, we believe our framework is transferrable to agricultural operations in other states and countries. It may support the improvement of indirect exposure assessment tools (e.g., surveys and questionnaires) and inform more comprehensive and appropriate direct observation methodologies to derive quantitative estimations of soil exposure in agriculture.

We recognize that the set of factors we identified may not be exhaustive and possess both quantitative and qualitative attributes that require further investigation. Given the qualitative nature of our data, our framework is intended to be used to guide the conduct of future studies designed specifically to quantify the magnitude, relative influence, or direction of each of these factors on soil exposure as well as to identify associations between soil exposure and health outcomes. Empirical data collected within this framework could also be used to inform an Agent-Based Model of Human Activity Patterns, which simulates longitudinal behaviors and exposures [39, 40]. Our framework also offers notable improvement in characterizing an agricultural worker’s comprehensive exposure by considering the potentially important medium of soil.

Estimation of soil exposure in the agricultural context is a key component necessary for the derivation of occupational health guidelines for soil contaminants, though agricultural workers are not the only population exposed to this medium. We believe our meso-activity approach is valid for generating soil contact estimates for other populations (e.g., children and adults in the general population, construction workers) who also have periodic soil contact. The purpose of the framework is to systematically identify qualitative factors for further investigation to characterize the direction and magnitude of their quantitative impact on soil exposure. As is, we do not intend or recommend the direct application of our framework to children’s play activities. Future studies may consider adaptations of our conceptual framework to collect additional qualitative and quantitative data on other exposure factors relevant to these populations and their specific meso-activities.

## Supplementary information

Supplementary Tables

Reporting Checklist

## Data Availability

Exposure calculations can be found in the supplementary information. Original transcripts of interviews can be obtained from the corresponding author.

## References

[1] Mielke HW, Anderson JC, Berry KJ, Mielke PW, Chaney RL, Leech M (1983). Lead concentrations in inner-city soils as a factor in the child lead problem. Am J Public Health.

[2] Riedo J, Wettstein FE, Rösch A, Herzog C, Banerjee S, Büchi L (2021). Widespread Occurrence of Pesticides in Organically Managed Agricultural Soils—the Ghost of a Conventional Agricultural Past?. Environ Sci Technol.

[3] Pepper IL, Brusseau ML, Prevatt FJ, Escobar BA (2021). Incidence of Pfas in soil following long-term application of class B biosolids. Sci Total Environ.

[4] Costello MCS, Lee LS. Sources, Fate, and Plant Uptake in Agricultural Systems of Per- and Polyfluoroalkyl Substances. Curr Pollution Rep. 2020. 10.1007/s40726-020-00168-y.

[5] Hubbard H, Özkaynak H, Glen G, Cohen J, Thomas K, Phillips L (2022). Model-based predictions of soil and dust ingestion rates for U.S. adults using the stochastic human exposure and dose simulation soil and dust model. Sci Total Environ.

[6] LaGoy PK (1987). Estimated soil ingestion rates for use in risk assessment. Risk Anal.

[7] US EPA. Exposure Factors Handbook 2011 Edition (Final Report). Washington, DC: U.S. Environmental Protection Agency; 2011. Report No.: EPA/600/R-09/052F.

[8] Lupolt SN, Agnew J, Burke TA, Kennedy RD, Nachman KE (2021). Key considerations for assessing soil ingestion exposures among agricultural workers. J Expo Sci Environ Epidemiol.

[9] Branco PT, Alvim-Ferraz MC, Martins FG, Sousa SI (2014). The microenvironmental modelling approach to assess children’s exposure to air pollution - A review. Environ Res.

[10] Adams MD, Yiannakoulias N, Kanaroglou PS (2016). Air pollution exposure: An activity pattern approach for active transportation. Atmos Environ.

[11] Burke JM, Zufall MJ, ÖZkaynak H (2001). A population exposure model for particulate matter: case study results for PM2.5 in Philadelphia, PA. J Expo Sci Environ Epidemiol.

[12] Ozkaynak H, Xue J, Zartarian VG, Glen G, Smith L (2011). Modeled estimates of soil and dust ingestion rates for children. Risk Anal.

[13] Zartarian VG, Streicker J, Rivera A, Cornejo CS, Molina S, Valadez OF (1995). A pilot study to collect micro-activity data of two- to four-year-old farm labor children in Salinas Valley, California. J Exposure Anal Environ Epidemiol.

[14] US EPA. Descriptive statistics from a detailed analysis of the National Human Activity Pattern Survey (NHAPS) responses. Washington, DC; 1996. Report No.: EPA/600/R-96/148.

[15] Garlock TJ, Shirai JH, Kissel JC (1999). Adult responses to a survey of soil contact-related behaviors. J exposure Anal Environ Epidemiol.

[16] Wang Y-L, Tsou M-CM, Pan K-H, Özkaynak H, Dang W, Hsi H-C (2021). Estimation of Soil and Dust Ingestion Rates from the Stochastic Human Exposure and Dose Simulation Soil and Dust Model for Children in Taiwan. Environ Sci Technol.

[17] Beamer PI, Canales RA, Bradman A, Leckie JO (2009). Farmworker children’s residential non-dietary exposure estimates from micro-level activity time series. Environ Int.

[18] Cohen Hubal EA, Sheldon LS, Burke JM, McCurdy TR, Berry MR, Rigas ML (2000). Children’s exposure assessment: a review of factors influencing Children’s exposure, and the data available to characterize and assess that exposure. Environ Health Perspect.

[19] Xue J, Zartarian V, Moya J, Freeman N, Beamer P, Black K (2007). A meta-analysis of children’s hand-to-mouth frequency data for estimating nondietary ingestion exposure. Risk Anal.

[20] Xue J, Zartarian V, Tulve N, Moya J, Freeman N, Auyeung W (2010). A meta-analysis of children’s object-to-mouth frequency data for estimating non-dietary ingestion exposure. J Expo Sci Environ Epidemiol.

[21] Ferguson A, Canales R, Vieira V, Leckie J (2013). Methodology to capture children’s non-dietary ingestion exposure activities during meal events. Hum Ecol Risk Assess.

[22] Beamer PI, Plotkin KR, Gerba CP, Sifuentes LY, Koenig DW, Reynolds KA (2015). Modeling of human viruses on hands and risk of infection in an office workplace using micro-activity data. J Occup Environ Hyg.

[23] Benke G, Sim M, Fritschi L, Aldred G (2000). Beyond the job exposure matrix (JEM): the task exposure matrix (TEM). Ann Occup Hyg.

[24] Selikoff IJ, Seidman H (1991). Asbestos-associated deaths among insulation workers in the United States and Canada, 1967-1987. Ann N. Y Acad Sci.

[25] Quinot C, Dumas O, Henneberger PK, Varraso R, Wiley AS, Speizer FE (2017). Development of a job-task-exposure matrix to assess occupational exposure to disinfectants among US nurses. Occup Environ Med.

[26] Coronado GD, Thompson B, Strong L, Griffith WC, Islas I (2004). Agricultural task and exposure to organophosphate pesticides among farmworkers. Environ Health Perspect.

[27] Dick FD, Semple SE, van Tongeren M, Miller BG, Ritchie P, Sherriff D (2010). Development of a Task-Exposure Matrix (TEM) for Pesticide Use (TEMPEST). Ann Occup Hyg.

[28] US EPA. Occupational Pesticide Handler Unit Exposure Surrogate Reference Table Washington, DC: U.S. Environmental Protection Agency; 2016 [updated November 2016. Available from: https://www.epa.gov/sites/production/files/2016-11/documents/handler-exposure-table-2016.pdf.

[29] Antwi-Agyei P, Biran A, Peasey A, Bruce J, Ensink J (2016). A faecal exposure assessment of farm workers in Accra, Ghana: a cross sectional study. BMC Public Health.

[30] Gale NK, Heath G, Cameron E, Rashid S, Redwood S (2013). Using the framework method for the analysis of qualitative data in multi-disciplinary health research. BMC Med Res Methodol.

[31] Saldaña J. The Coding Manual for Qualitative Researchers. 3rd ed. London: Sage Publications; 2016.

[32] Zartarian V, Bahadori T, McKone T (2005). Adoption of an official ISEA glossary. J exposure Anal Environ Epidemiol.

[33] US EPA. Risk Assessment Guidance for Superfund, Volume I - Human Health Evaluation Manual. 1989.

[34] Irwin A, Mihulkova J, Berkeley S, Tone LR (2022). ‘No-one else wears one:’ Exploring farmer attitudes towards All-Terrain Vehicle helmets using the COM-B model. J Saf Res.

[35] Trenerry C, Fletcher C, Wilson C, Gunn K (2022). “She’ll Be Right, Mate”: A Mixed Methods Analysis of Skin Cancer Prevention Practices among Australian Farmers-An At-Risk Group. Int J Environ Res Public Health.

[36] Shwe S, Sharma AA, Lee PK (2021). Personal Protective Equipment: Attitudes and Behaviors Among Nurses at a Single University Medical Center. Cureus.

[37] Parks CG, Meyer A, Beane Freeman LE, Hofmann JN, Sandler DP (2019). Farming tasks and the development of rheumatoid arthritis in the agricultural health study. Occup Environ Med.

[38] US EPA. Revised Risk Assessment Methods for Workers, Children of Workers in Agricultural Fields and Pesticides with No Food Uses. In: Programs OoP, editor. Washington, DC 2009. p. 10.

[39] Brandon N, Price PS (2019). Calibrating an agent-based model of longitudinal human activity patterns using the Consolidated Human Activity Database. J Expo Sci Environ Epidemiol.

[40] Brandon N, Dionisio KL, Isaacs K, Tornero-Velez R, Kapraun D, Setzer RW (2018). Simulating exposure-related behaviors using agent-based models embedded with needs-based artificial intelligence. J Expo Sci Environ Epidemiol.

